# A BAC Transgene Expressing Human CFTR under Control of Its Regulatory Elements Rescues Cftr Knockout Mice

**DOI:** 10.1038/s41598-019-48105-4

**Published:** 2019-08-14

**Authors:** Lara R. Gawenis, Craig A. Hodges, Daniel R. McHugh, Dana M. Valerio, Alexander Miron, Calvin U. Cotton, Jinghua Liu, Nancy M. Walker, Ashlee M. Strubberg, Austin E. Gillen, Michael J. Mutolo, George Kotzamanis, Jürgen Bosch, Ann Harris, Mitchell L. Drumm, Lane L. Clarke

**Affiliations:** 1 Dalton Cardiovascular Research Center, University of Missouri, 134 Research Park Dr, Columbia, Missouri 65211-3300 USA; 2 Department of Biomedical Sciences, University of Missouri, E102 Veterinary Medicine Bldg., Columbia, Missouri 65211 USA; 3 Departments of Pediatrics, Case Western Reserve University School of Medicine, Cleveland, Ohio USA; 4 Departments of Physiology and Biophysics, Case Western Reserve University School of Medicine, Cleveland, Ohio USA; 5 Departments of Genetics and Genome Sciences, Case Western Reserve University School of Medicine, Cleveland, Ohio USA; 6 Human Molecular Genetics Program, Lurie Children’s Research Center, Northwestern University Feinberg School of Medicine, Chicago, IL 60614 USA; 7 Department of Histology and Embryology, School of Medicine, University of Athens, Athens, Greece; 8 InterRayBio, LLC, Baltimore, MD USA; 9 Present Address: RNA Bioscience Initiative, University of Colorado School of Medicine, Aurora, CO USA

**Keywords:** Cystic fibrosis, Gastrointestinal models, Experimental models of disease

## Abstract

Small-molecule modulators of cystic fibrosis transmembrane conductance regulator (CFTR) biology show promise in the treatment of cystic fibrosis (CF). A Cftr knockout (Cftr KO) mouse expressing mutants of human CFTR would advance *in vivo* testing of new modulators. A bacterial artificial chromosome (BAC) carrying the complete *hCFTR* gene including regulatory elements within 40.1 kb of DNA 5′ and 25 kb of DNA 3′ to the gene was used to generate founder mice expressing hCFTR. Whole genome sequencing indicated a single integration site on mouse chromosome 8 (8qB2) with ~6 gene copies. hCFTR+ offspring were bred to murine Cftr KO mice, producing hCFTR+/mCftr− (H+/m−) mice, which had normal survival, growth and goblet cell function as compared to wild-type (WT) mice. Expression studies showed hCFTR protein and transcripts in tissues typically expressing mCftr. Functionally, nasal potential difference and large intestinal short-circuit (I_sc_) responses to cAMP stimulation were similar in magnitude to WT mice, whereas small intestinal cAMP ΔI_sc_ responses were reduced. A BAC transgenic mouse with functional hCFTR under control of its regulatory elements has been developed to enable the generation of mouse models of hCFTR mutations by gene editing for *in vivo* testing of new CF therapies.

## Introduction

High throughput screening and lead optimization have led to the successful introduction of FDA-approved small molecular compounds that modulate the biology of the cystic fibrosis transmembrane conductance regulator (CFTR) for the treatment of cystic fibrosis (CF) patients. Greatest strides have been made with “potentiator” compounds, e.g., Ivacaftor®, that increase CFTR channel gating, likely through direct binding to the mutant protein^1^. CF patients that benefit the most have type III mutations of at least one allele where CFTR protein is made in sufficient amounts and traffics to the plasma membrane but has defective channel gating, e.g., G551D. Some with type IV mutations, i.e., defective channel conduction (e.g., R117H) also benefit from potentiator treatment. Less efficacious have been the “corrector” compounds, e.g. Lumacaftor®, that function to chaperone the mutant protein during cellular processing. Correctors are targeted at CFTR mutations, including ΔF508 which constitutes ~85% of CFTR mutant alleles^1^ (https://www.cff.org/Research/Developing-New-Treatments/CFTR-Modulator-Types/). The success of these compounds have spawned great interest in the development of the next generation of CFTR modulators, including “amplifiers” that increase the CFTR expression, and other therapeutic modalities such as targeting sodium channel activity or mucus secretion. Therefore, the development of mouse models expressing human CFTR (hCFTR) and relevant mutations in the absence of endogenous murine Cftr (mCftr) may be useful for pre-clinical *in vivo* trials to assess new modulator compounds.

Beyond heterologous expression systems, stable hCFTR expression in murine tissues has previously been accomplished using a yeast artificial chromosome (YAC) to rescue mCftr knockout mice^2,3^. A 310 kb YAC genomic construct containing the entire coding region of hCFTR and ~70 kb upstream of the 5′ end of exon 1 was used in pronuclear injection to generate transgenic mice expressing hCFTR. The two clones of YAC hCFTR mice were bred to the cftr^tm1CAM^ null allele mice to complement the loss of endogenous mCftr^2^. Both clones increased survival and showed functional expression of hCFTR in intestinal segments, with one clone showing wild-type responses to cAMP stimulation of anion secretion and the other showing lower responses. Only one clone corrected cAMP-stimulated chloride secretion in the mCftr-null trachea. Overall, the YAC hCFTR matched mCftr tissue-specific expression in intestines and salivary glands, and low expression in the lung and pancreas. At the time, molecular studies of CFTR expression identified some DNase I hypersensitive (DHS) sites responsible for tissue-specific expression and tissue-specific enhancer activity in the h*CFTR* locus, which were also evident in the YAC h*CFTR*^4,5^. Work that is more recent identified multiple tissue specific DHS containing critical regulatory elements both 5′ to the promoter and 3′ to the end of the gene in airway epithelial cells^6,7^ and within introns in intestinal epithelial cells^8^. Based on the earlier studies and interest in the development of a gene therapy construct, a 250.3 kb bacterial artificial chromosome (BAC) transgene was engineered containing the entire coding region of hCFTR, 40.1 kb of DNA 5′ and 25 kb of DNA 3′ to the gene^9^. Though this BAC lacks the topologically associating domain (TAD) boundaries for *CFTR* (at −80.1 kb and +48.9 kb^10–12^) it contains the majority of key tissue-specific control elements for the locus. In the present study, this construct was used to develop a BAC transgenic mouse model expressing hCFTR by its endogenous regulatory elements with the purpose of introducing different CF mutations by gene editing and subsequent *in vivo* testing of CFTR modulators and other therapeutic modalities for the treatment of cystic fibrosis.

## Results

### BAC hCFTR transgenic mice

Nine founders were produced from pronuclear injection (4 females, 5 males). Of the nine, 3 male founders produced pups that were transgene positive. Jejunal tissue from the offspring were tested for intact hCFTR mRNA using a TaqMan® (Life Sciences) assay directed at the exon 27–28 boundary of hCFTR. Only the offspring from one male founder was strongly positive for hCFTR mRNA at this site. Offspring from the hCFTR founder were bred into mCftr KO mice to generate hCFTR+/mCftr− (H+/m−) mice.

### Integration site and copy number of the hCFTR BAC

**Figure 1:**
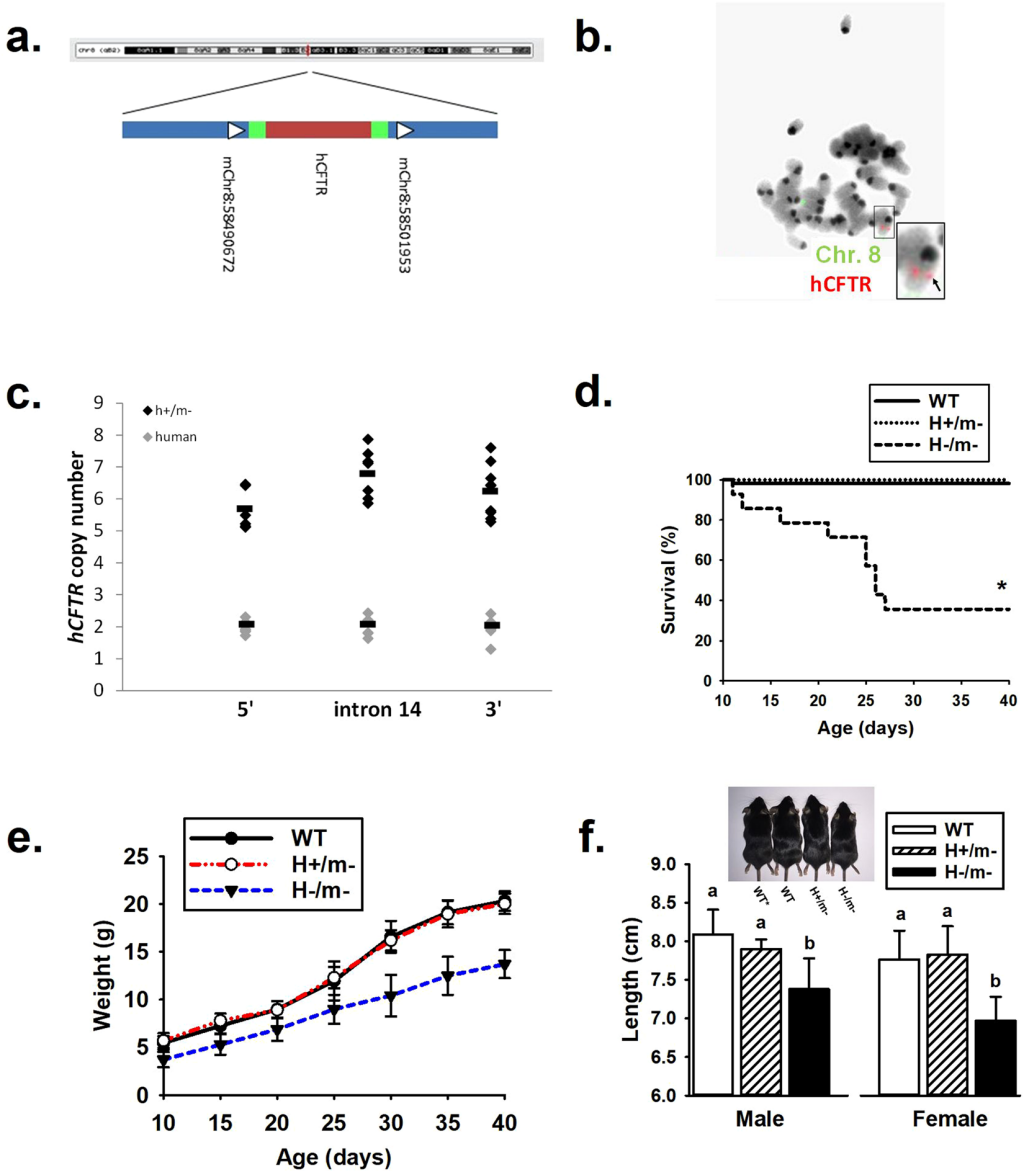
Rescue of mCftr KO mouse with hCFTR BAC transgene. (**a**) Integration locus: hCFTR BAC transgene inserted into a single insertion site on mouse chromosome 8qB2 (indicated by the red line). Region is expanded to show the exact site in chromosome 8 region with arrows indicating directionality, green bars indicating BAC backbone and red indicating hCFTR (not to scale). (**b**) Fluorescence *in situ* hybridization using a fluorescently labeled BAC (red) and a distal chromosome 8-labeled probe (green) on embryonic stem cells from a H+/m− mouse embryo. (**c**) qPCR analysis of hCFTR copy number to three regions of the hCFTR BAC (5′ of hCFTR, intron 14 and 3′ of hCFTR) in H+/m− mice (black circles) and human (gray circles) control DNA samples. (**d**) Survivability of H/m genotypes. (n ≥ 14 mice/genotype). (**e**) Body weight of H/m genotypes during first 40 days of age (n = 20 per group with equal numbers of each sex). Bars indicate standard deviation. (**f**) Body length comparison of H/m genotypes by sex (n ≥ 6 per group). Inset, comparison of body length by genotype, inclusive of a non-colony B57BL/J wild-type mouse (WT*). ^a,b^Means with the same letters are not significantly different.

DNA from an hCFTR+ mouse was isolated and whole genome sequencing was completed. A single integration site was identified on mouse chromosome 8 in region 8qB2 (Fig. 1a). Using UCSC Genome Browser®, we observed no promoters, enhancers or heterochromatin in this region that would significantly impact expression of the hCFTR transgene. The closest promoter for a gene is over 400 kb away from the integration site. The single insertion site was verified with a fluorescently labeled BAC in mouse embryonic stem cells from an hCFTR+ mouse (Fig. 1b). To determine the number of copies of the hCFTR BAC integrated at this single site, qPCR was completed at three sites within the hCFTR BAC sequence. Compared to genomic human DNA (2 copies), there was an average of 5.6, 6.8 and 6.2 copies 5′, within hCFTR and 3′ of the gene respectively (Fig. 1c).

### Survivability and growth of hCFTR mice

Post-weaning cohorts of WT, H+/m− and hCFTR−/mCftr− (H−/m−) were provided standard mouse chow and regular drinking water to determine survivability. Body weight and length were also measured. As shown in Fig. 1d, all but one of the WT mice and all H+/m− mice survived the post-weaning period until terminated at 40 days of age. In contrast, H−/m− mice had reduced survivability (~40%) during the same time period. Of the surviving mice (Fig. 1e), both WT and H+/m− mice gained body weight at a rate of 0.50 g/day which compares favorably to previous reports of WT mouse growth rates (0.53 g/day) of mice during the period of 15 to 30 days of age^13^. As expected^13^, H−/m− mice during this time period grew at a slower rate (0.13 g/day). As shown in Fig. 1f, body length of the mice also reflected differences in growth with nearly equivalent body length between WT C57Bl/6 (Jackson Labs), WT from the colony and H+/m− mice, with all significantly exceeding the body length of H−/m− mice.

### Tissue-specific hCFTR mRNA expression

**Figure 2:**
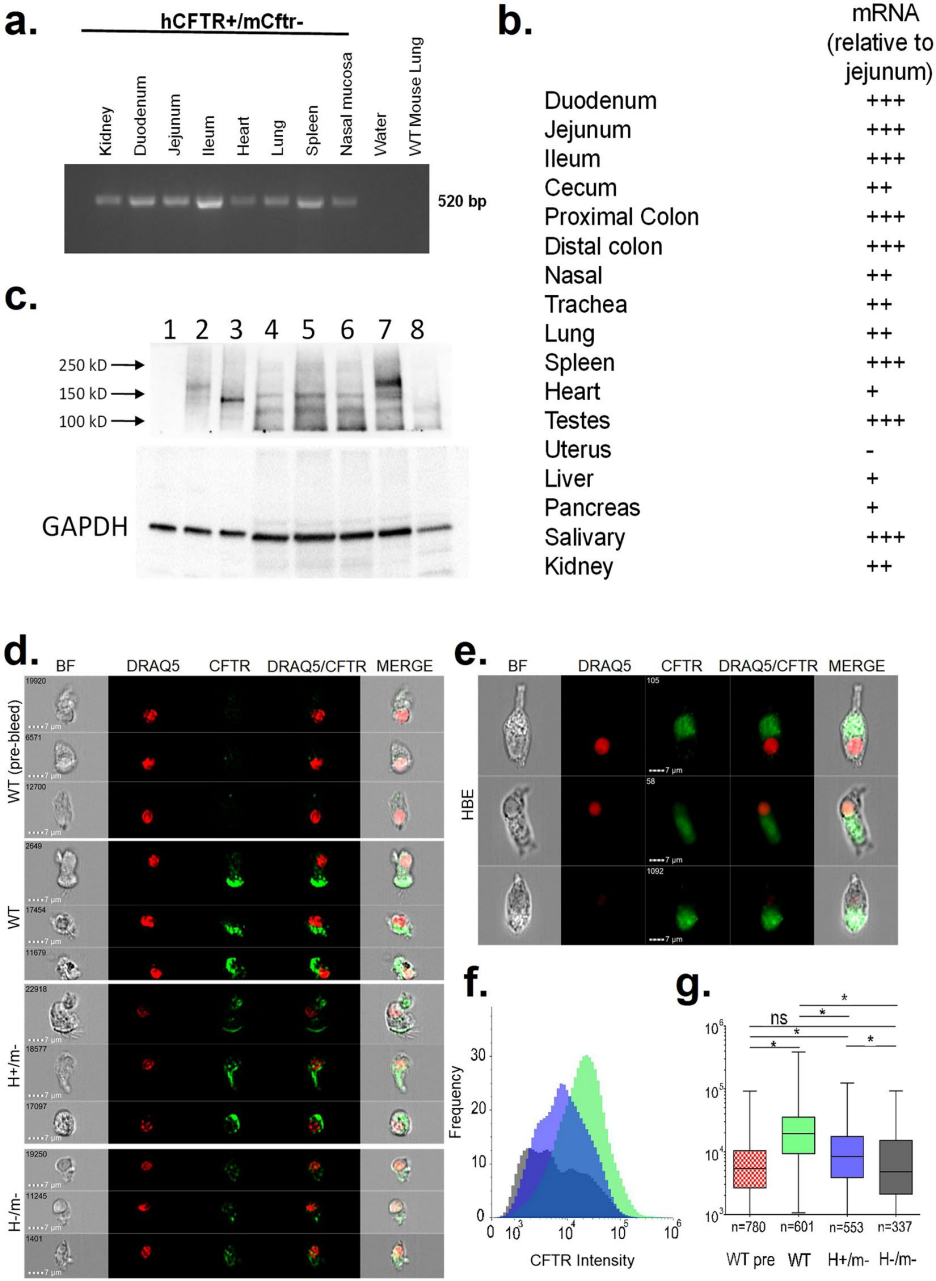
Tissue-specific expression. (**a**) Agarose gel displaying mRNA tissue specific expression of hCFTR in H+/m− mice. (**b**) qPCR results displaying relative tissue-specific mRNA expression of hCFTR in H+/m− mice. (n = 3–6 mice). (**c**) Immunoblot analysis of recombinant hCFTR expressed in CHO cells, hCFTR in freshly isolated crypts from three H+/m− mice and mCftr WT and KO intestine. Lane 1, untransfected CHO cells; Lane 2, recombinant hCFTR transfected CHO cells with sham treatment; Lane 3, recombinant hCFTR CHO cells treated with N-glycanase; Lane 4–6, H+/m− jejuna from 3 H+/m− mice; Lane 7, freshly-isolated crypts from a WT mouse and Lane 8, freshly-isolated crypts from a Cftr KO jejunum. Arrows indicate molecular mass markers. Lower panel, GAPDH as loading control. Representative of 9 H+/m− mice. (**d**) Imaging flow cytometry analysis of mouse tracheal epithelial cells stained with anti-CFTR RB7865 and the nuclear stain DRAQ5. Representative cells from WT, H+/m− and H−/m− murine cells in brightfield (BF), stained for DRAQ5 (red) and CFTR (green), and merged images. The top panel of images represents WT cells stained with the pre-immune isotype control of RB7865. Second through fourth panels represent WT, H+/m− and H−/m− airway cells, respectively. (**e**) Representative human bronchial epithelial cells. Note the localization of the CFTR signal towards the cilia in both panels. (**f**) Population quantification of all selected and analyzed mouse tracheal cells displaying the frequency of a cell with each intensity for the antibody with WT pre-bleed (red), WT (green), H+/m− (blue) and H−/m− (grey). (**g**) Overall CFTR intensity for each population. The mean and 95% confidence level is represented in the shaded box, whiskers indicate minimum and maximum in the dataset. Numbers below each sample set represent the final amount of cells used for analysis. *Significantly different from each other.

Using qRT-PCR, the tissue-specific expression of the hCFTR transgene was screened in several tissues of H+/m− mice. Figure 2a,b shows strong expression in intestinal segments of the small and large bowel. High expression was also noted in the submandibular salivary glands, spleen and the testes. hCFTR was expressed in other tissues affected by CF disease including the airways (nasal, trachea, lung), liver and pancreas. Little or no expression was apparent in the uterus. The enamel organ of the incisor teeth was not tested but H+/m− mice exhibited white central incisors with chipping of the tooth edges on the lower incisor pair. This is an overt phenotypic characteristic of Cftr KO mice as a result of reduced mineralization and low Fe levels in the enamel of the hypsodont incisors^14^.

### hCFTR protein expression in H+/m− in intestine and airways

Immunoblots for hCFTR in the mCftr- background in isolated small intestinal crypts were compared to CHO cells expressing recombinant human CFTR (a kind gift from T.C. Hwang, University of Missouri). N-glycanase treatment of the cells enabled assessment of the glycosylation status of hCFTR^15^. As shown in Fig. 2c (lane 2), recombinant hCFTR expressed in CHO cells migrated primarily as the fully glycosylated band C and a small amount of core glycosylated protein (band B), migrating at ~175 kD and ~150 kD, respectively. After treatment with N-glycanase, the recombinant hCFTR migrated primarily as band A  (lane 3). Immunoblot analysis of hCFTR in freshly-isolated intestinal crypts from three H+/m− mice showed a band migrating near the region of band A (~135 kD) as indexed by the N-glycanase treated recombinant CFTR  (lanes 4-6). Murine Cftr showed band C staining (lane 7) whereas a banding pattern for Cftr was not detected in isolates from Cftr KO intestinal crypts  (lane 8), indicating the specificity of the anti-CFTR 3g11 antibody. All mouse intestinal isolates also showed a non-specific band migrating at ~125 kD. N-glycanase treatment of H+/m− intestinal isolates did not affect the migration pattern of the hCFTR-specific band (data not shown). CFTR protein expression was also assessed in lung epithelial cells using imaging flow cytometry. CFTR presence is noted on the apical ciliated end of the lung epithelial cells from WT and H+/m− but not from H−/m− mice (Fig. 2d). A similar staining pattern was observed in human bronchial epithelial cells (Fig. 2e). While there is some nonspecific staining in H−/m− cells, the number and intensity of CFTR staining in WT and H+/m− cells is significantly different than in H−/m− cells (Fig. 2f,g).

### Intestinal morphology of the hCFTR mice

**Figure 3:**
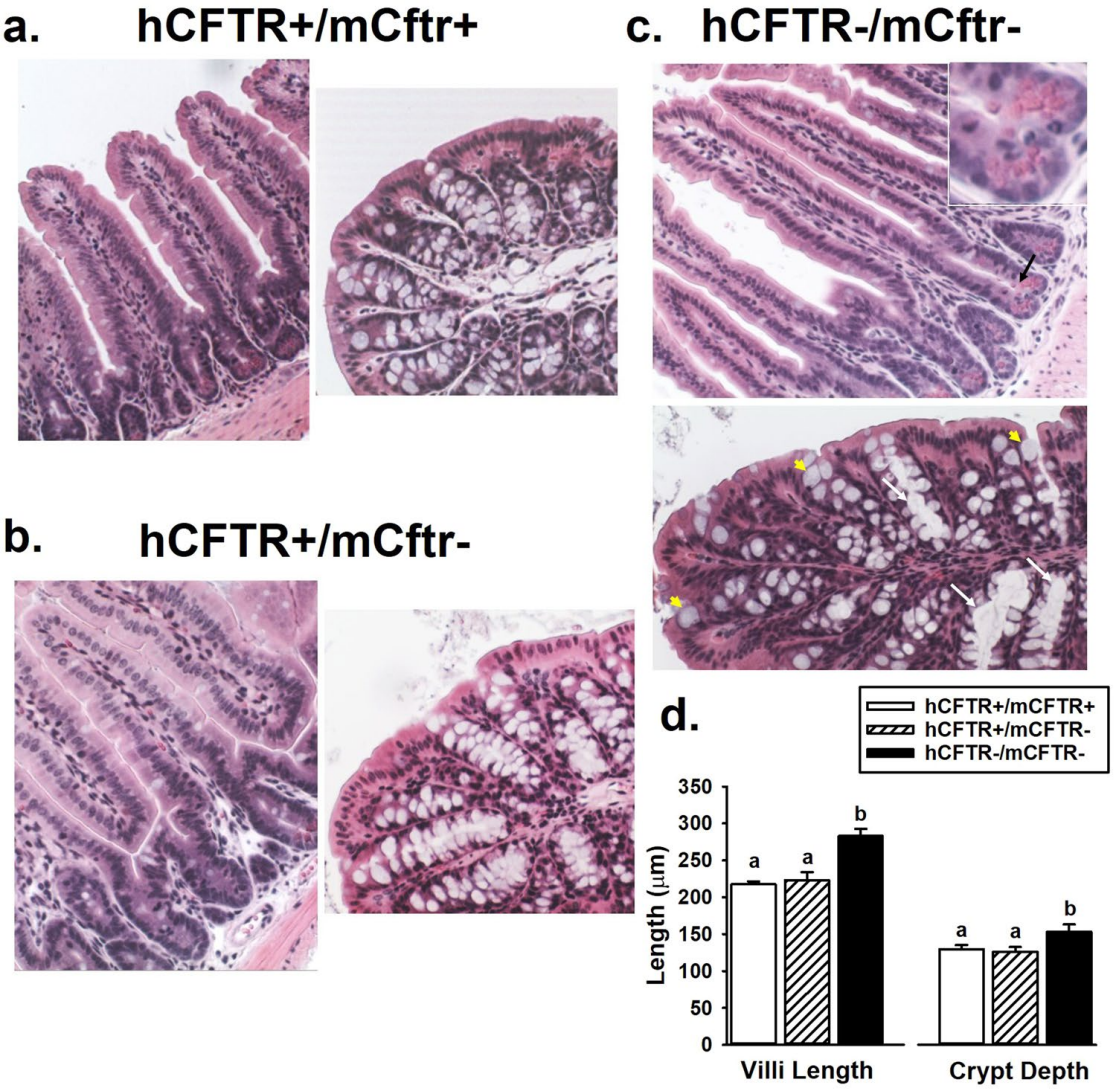
Intestinal morphology. Representative hematoxylin/eosin-stained sections of the jejunum and proximal colon from (**a**) WT (H+/m+), (**b**) H+/m− and (**c**) H−/m− mice (200X mag). In the H−/m− sections, mucus-encased Paneth cell granules are shown with black arrow and inset; enlarged goblet cells are indicated by yellow arrowheads; and crypt plugging is indicated by white arrows. Representative of n = 4 mice of each genotype. (**d**) Villus and crypt lengths in H/m genotypes, n = 5 mice/genotype. ^a,b^Means with the same letters are not significantly different.

Intestinal pathology is the major manifestation of cystic fibrosis in Cftr KO mouse models. Intestinal pathogenesis involves mucoviscidosis that includes goblet cell hyperplasia, increased goblet cell volume, viscid mucus and mucus plugging of crypts. Another morphological difference of Cftr KO mouse intestine is increased length of the crypt-villus axis resulting from increased epithelial proliferation and, possibly, decreased apoptosis^16,17^. Histological sections of the jejunum and proximal colon were compared among WT, H+/m− and H−/m− mice. As shown Fig. 3a,b, the appearance of the jejunum and the proximal colon was indistinguishable between WT and H+/m− mice. In contrast, the H−/m− jejunum showed overt increases in the length of the crypt-villus axis (see quantification in Fig. 3d). Furthermore, despite the mitigating effect of osmotic laxative treatment^18^, both the jejunum and proximal colon of H−/m− mice showed evidence of mucoviscidosis including mucus plugging of crypts mucus, trapping of Paneth cell granules and enlarged goblet cells, as shown in Fig. 3c and inset.

### Goblet cell exocytosis in the hCFTR intestine

**Figure 4:**
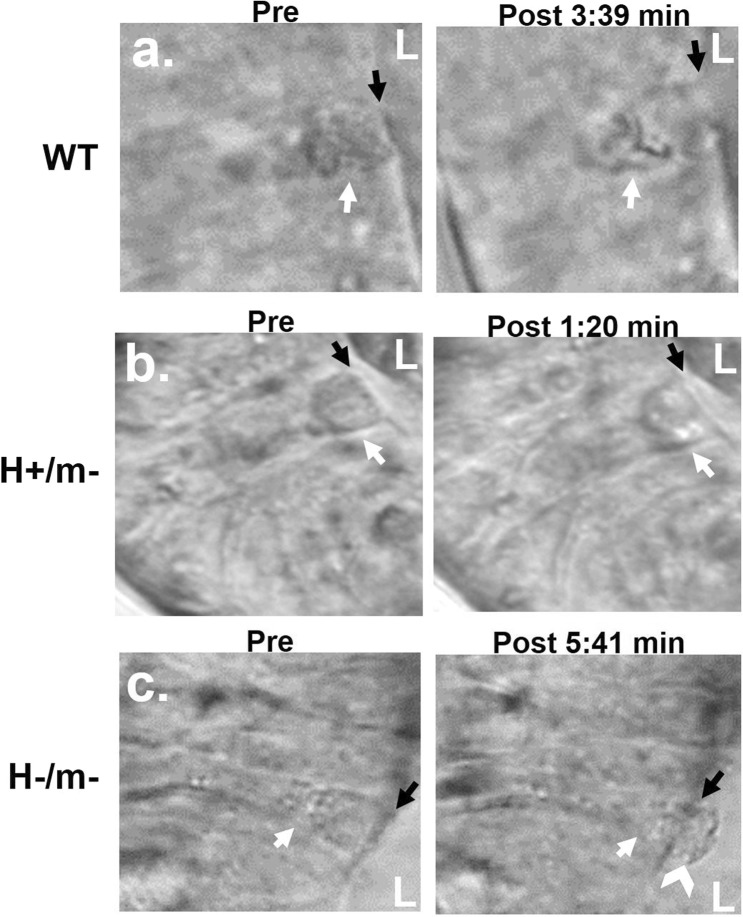
Effect of hCFTR on goblet cell degranulation in intestinal organoids. (**a**) WT goblet cell (white arrow) in distal jejunum undergoes rapid exocytosis from the apical pole of the theca with dissolution of granules. L, lumen of organoid. Black arrow, denotes apical (luminal) membrane. (**b**) H+/m− goblet cell (white arrow) also undergoes rapid, normal exocytosis. L, lumen of organoid. Black arrow, denotes apical (luminal) membrane. (**c**) H−/m− goblet cell (white arrow) undergoes slow exocytosis with mucus anchoring to goblet cell theca (white chevron). Black arrow, denotes apical (luminal) membrane. Representative of 3 experiments (Mag. 600x).

Mucus anchoring, whereby expelled mucus from Cftr KO goblet cells remains attached at the goblet cell theca, is a contributing factor to mucoviscidosis and intestinal obstruction by expanding the surface area for mucus attachment to the epithelial surface^19^. Videomicroscopy was used to evaluate goblet cell exocytosis during carbachol stimulation in cultured distal jejunal organoids from sex-matched WT, H+/− and H−/m− littermate mice. As shown in Fig. 4a, goblet cell exocytosis induced by carbachol treatment in organoids from WT mice resulted in normal degranulation as indicated by immediate dissolution of released granules and “cupping” at the apical theca of the partially degranulated goblet cell. Similarly, goblet cells in H+/m− organoids underwent rapid and apparently normal exocytosis (Fig. 4b). In contrast, exocytosis in H−/m− goblet cells was slower and resulted in the formation of anchored mucus plugs extending into the intestinal lumen (Fig. 4c).

### Functional activity of hCFTR in murine epithelia

**Figure 5:**
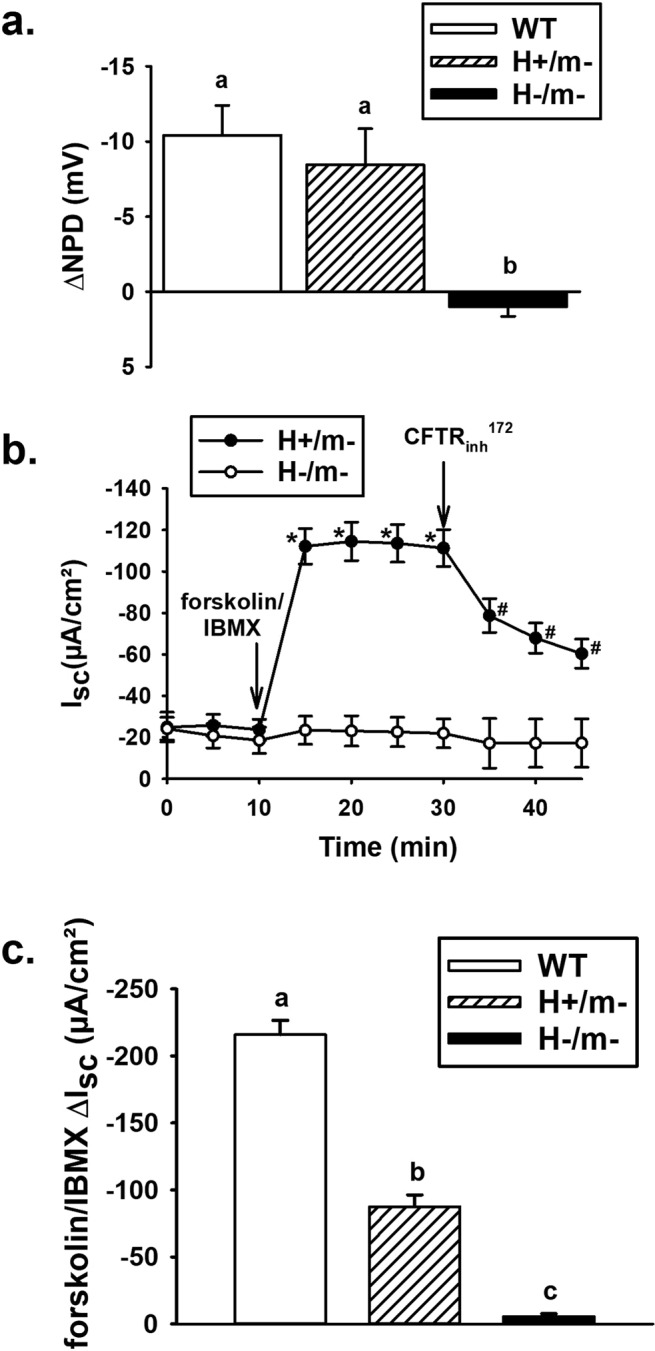
Functional activity of hCFTR in nasal and intestinal mucosa. (**a**) Change in nasal potential difference (NPD) after forskolin stimulation in a chloride-free solution (n ≥ 6 mice/genotype). (**b**) Short-circuit current (I_sc_) responses of H+/m− and H−/m− jejuna to forskolin (10 µM)/IBMX (100 µM) stimulation and subsequent treatment with CFTR_inh_-172 (10 µM), n = 10 and 4 mice, respectively. Only two of four H−/m− mice were treated with CFTR_inh_-172. *Significantly different from time 0. ^#^Significantly different from time 20 min. (**c**) Cumulative data for forskolin/IBMX ΔI_sc_ responses of WT (n = 3 mice), H+/m− (n = 10 mice, from Fig. 4D) and H−/m− (n = 3 mice) jejunal sections. ^a,b,c^Means with the same letters are not significantly different.

Activation of CFTR and Cl^−^ secretion into the nasal lumen causes hyperpolarization of the nasal epithelium. As shown in Fig. 5a, *in vivo* measurements of cAMP-stimulated nasal potential difference (ΔNPD)^20^ showed similar magnitude of responses between WT and H+/m− mice; whereas H−/m− mice exhibited a ΔNPD that was not significantly different from zero (1 ± 0.64 mV).

Intestinal sections were mounted in Ussing chambers for the measurement of short-circuit current (I_sc_), as previously described^21^. As shown in Fig. 5b, H+/m− jejunal sections had a robust I_sc_ response to intracellular cAMP stimulation within 5 min, which was ~45–50% inhibited within 15 min by CFTR_inh_-172. In contrast, the H−/m− jejunum had virtually no response to cAMP stimulation or treatment with CFTR_inh_-172. A comparison of the basal and stimulated electrophysiological properties of the jejunum from WT, H+/m− and H−/m− mice are shown in Table 1. Under basal conditions, WT jejuna have a significantly greater basal I_sc_ as compared to the H+/m−and H−/m− jejuna, which were not statistically different from each other. Transepithelial conductance (G_t_), primarily a measure of paracellular ion permeability of the small bowel^22^, was not different among the genotypes. Following cAMP stimulation using a forskolin/IBMX cocktail, the I_sc_ increased significantly in all genotypes except the H−/m− intestine. However, the cAMP-stimulated ΔI_sc_ was greater in WT as compared the H+/m− intestine (shown graphically in Fig. 5c). No significant differences in G_t_ between genotypes were detected after cAMP stimulation.

**Table 1:** Electrophysiology of proximal jejunum.

	WT		H+/m−		H−/m−	
	I_sc_	G_t_	I_sc_	G_t_	I_sc_	G_t_
**Genotype**						
Basal	−40.9^*^	38.5	−15.1	40.3	−14.3	34.0
	±6.1	±4.0	±4.8	±2.6	±6.5	±3.5
cAMP	−252.1^+,*^	33.9	−99.9^+^	40.8	−17.0^*^	35.5
	±20.9	±5.4	±9.8	±2.8	±8.3	±4.7
	(6)		(10)		(3)	

I_sc_, short-circuit current (µA/cm²).

G_t_, transepithelial conductance (mS/cm²).

*P < 0.05 vs. H+/m−; ^+^P < 0.05 vs. Basal.

Parentheses = number of mice.

### Comparison of I_sc_ between H+/m− and WT mice in different intestinal segments

**Figure 6:**
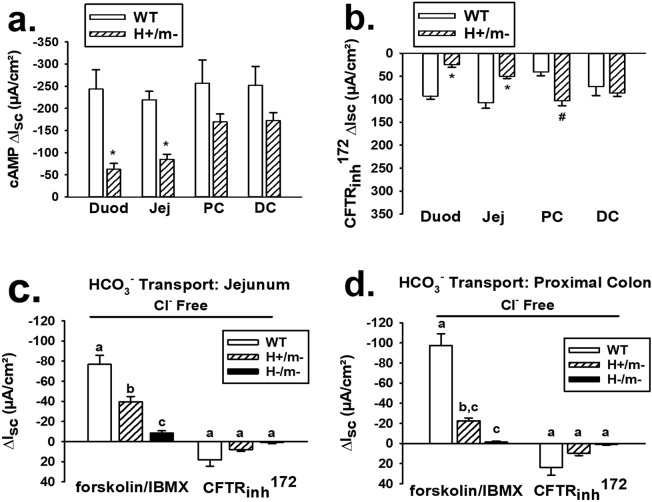
Comparison of function between hCFTR and mCftr in different intestinal segments and bicarbonate (HCO_3_^−^) transport by hCFTR in mouse jejunum and colon. (**a**) Comparison of change in short-circuit current (ΔI_sc_) to cAMP stimulation by forskolin (10 µM)/IBMX (100 µM) in duodenum (Duod), jejunum (Jej), proximal colon (PC) and distal colon (DC) mucosae between WT and H+/m− sex-matched littermate mice (n = 6 mice of each genotype). *Significantly less than WT. (**b**) Effect of CFTR_inh_-172 on ΔI_sc_ after 20-min forskolin/IBMX treatment in different intestinal segments comparing H+/m− and WT sex-matched littermate mice (n = 6 mice of each genotype). *Significantly less than WT. ^#^Significantly greater than WT. (**c**,**d**) ΔI_sc_ response to forskolin/IBMX and subsequent treatment with CFTR_inh_-172 superfused with Cl^–^free KBR solution in jejunum and proximal colon, respectively, n = 6 WT and H+/m− mice; n = 3 H−/m− sex-matched littermate mice. ^a,b,c^Means with the same letters are not significantly different.

Based on finding a lower cAMP ΔI_sc_ response in H+/m− jejunum as compared to that in WT mice, we investigated the cAMP- and Cftr_inh_-172-induced I_sc_ responses in different intestinal segments between sex-matched H+/m− and WT littermate mice. As shown in Fig. 6a, the cAMP-induced I_sc_ in H+/m− mice was significantly less in the duodenum and jejunum, as compared to that in the WT mice. However, the cAMP-induced I_sc_ in the large bowel (proximal and distal colon) was comparable between the two genotypes and did not attain a statistically significant difference. As shown in Fig. 6b, inhibition of the cAMP-stimulated I_sc_ using CFTR_inh_-172 was greater in the WT small intestine, but owing to the greater cAMP-induced I_sc_, the percent inhibition by CFTR_inh_-172 was similar between H+/m− and WT intestine, respectively (Duodenum %ΔI_sc_ = −45.3 ± 8.2 vs. −38.5 ± 4.2, ns; Jejunum %ΔI_sc_ = −51.6 ± 5.4 vs. −43.5.6 ± 5.0, ns). In the large intestine, inhibition of the cAMP-stimulated I_sc_ by CFTR_inh_-172 was significantly greater in the H+/m− proximal colon and similar in the distal colon, as compared to WT colon.

### Bicarbonate transport by hCFTR intestinal epithelium

CFTR is permeable to the anions Cl^−^ and HCO_3_^−^ at a ratio of approximately 4:1^23^. To assess HCO_3_^−^ transport by hCFTR, jejunal and proximal colonic sections from H+/m−, WT and H−/m− mice were compared for I_sc_ responses to a forskolin/IBMX cocktail while bathed in Cl^−^ free KBR. As shown in Fig. 6c, cAMP stimulation significantly increased the I_sc_ in the H+/m− jejunum relative to that in the H−/m− jejunum by ~30 µA/cm², which equates to a secretory HCO_3_^−^ flux of ~1 µeq/cm²·hr. A greater ΔI_sc_ response was exhibited by the WT jejunum which exceeded the I_sc_ response by the H−/m− jejunum by ~60 µA/cm², equating to a secretory HCO_3_^−^ flux of ~2 µeq/cm²·hr. Subsequent treatment with CFTR_inh_-172 had a negligible inhibitory effect on the HCO_3_^−^dependent I_sc_ in the three genotypes. A similar pattern to cAMP stimulation and CFTR inhibition emerged in studies of the proximal colon (Fig. 6d), although the H+/m− Cl^−^ free ΔI_sc_ response to cAMP stimulation did not attain statistical significance in comparison to that of the H−/m− proximal colon. In contrast, the ΔI_sc_ response to forskolin/IBMX was more robust in the WT colon and significantly exceeded the response by the H+/m− colon, yielding a calculated HCO_3_^−^ flux of ~3.5 µeq/cm²·hr. Subsequent treatment with CFTR_inh_-172 had little effect on the ΔI_sc_ response in the three genotypes.

## Discussion

The goal of this research was to develop a BAC transgenic mouse model expressing genomic human CFTR under control of its native regulatory elements. The value of this model is the possibility of using CRISPR/Cas9 gene editing to generate H+/m− mice expressing common *CFTR* mutations at the same integration site. Thus, CFTR modulators and other treatments can be compared in a consistent genetic background *in vivo* in mice.

Two early manifestations of CF disease are bowel obstruction and growth retardation. Intestinal obstructive disease in CF includes a spectrum from meconium ileus/meconium stasis at birth to obstructive syndromes in adult CF patients^24,25^. Growth abnormalities include reduced height and weight for age and percentage of ideal body weight^26^. Both bowel obstruction and growth retardation are recapitulated in CF mouse models^14,27,28^. Further, studies of enterocyte-specific knockout and knock-in of mCftr indicate that non-epithelial tissues also contribute to both growth retardation and bowel obstruction^29^. The lack of bowel obstruction and growth retardation in H+/m− mice is evidence of regulated hCFTR expression in the BAC transgenic model. One apparent exception is the enamel organ because the phenotype of white teeth was not corrected by  the hCFTR transgene. However, rodents have continuously growing hypsodont incisors, whereas all dentition in humans are slow-growing brachydont teeth. Brachydont molars in mice require little or no Cftr activity for normal mineralization^14^. Fortuitously, the white incisor teeth phenotype provides an overt indicator of a mCftr-null background in the hCFTR mice.

Consistent with regulated hCFTR expression, screening for hCFTR mRNA transcripts showed expression in various tissues known to express endogenous mCftr. Strongest expression was in the salivary glands, intestinal tract and testes, a finding consistent with human studies^30–32^. Based on our functional tests, the hCFTR mouse appeared to have an increasing proximal-distal gradient of CFTR activity whereas *in situ* hybridization studies in human show a decreasing proximal-distal intestinal gradient of CFTR expression^31^. hCFTR expression was present in the airways but at lower levels, as reported in human^7^. Most mRNA was collected from whole organ homogenates and not epithelial isolations, thus in CF-affected organs like pancreas and liver^32^, low expression is likely due to the dilution of the ductal hCFTR mRNA by RNA from the organ parenchyma.

The CFTR immunoblot of the H+/m− jejunal lysates indicated that hCFTR protein in the H+/m− intestine appears to migrate as band A, indicating that hCFTR is unglycosylated. This observation is supported by inability of N-glycanase to affect the hCFTR band migration and specificity of the antibody by lack of the band in the Cftr KO mouse intestine. Since six copies of the BAC transgene were detected at the integration site, it is possible that sufficient amounts of unprocessed hCFTR localized to the apical membrane to provide the robust functional activity shown in the electrophysiological experiments. In support, previous studies have shown that overexpression of the poorly glycosylated ΔF508 CFTR in cell systems yields cAMP-stimulated halide efflux^33^. Sequencing at both ends of the hCFTR insertion site indicated that the integration site was adjacent to the backbone of the BAC on both sides, but it is possible that most of the hCFTR copies rearranged to yield either misfolded protein or a configuration that interfered with the glycosylation process. Understanding the changes in glycosylation pattern of the hCFTR will likely require complete sequencing of the integration site.

Pathological features of intestinal CF that are modeled by Cftr KO mice include mucoviscidosis and a hyperproliferative intestinal mucosa^17,27,34^. Morphological assessment of the H+/m− jejunum and proximal colon did not reveal overt differences from tissue sections from WT mice. As expected, H−/m− small intestine showed evidence of mucoviscidosis, goblet cell dysfunction and increased length of the crypt-villus axis. Thus, expression of the hCFTR transgene in the mCftr- mouse corrects two disparate manifestations of intestinal CF that have been associated with obstructive bowel disease and an increased risk of gastrointestinal cancer in CF patients^27,34^.

Cyclic AMP stimulation of anion secretion by the hCFTR transgene was observed in the mCftr-null background in both airway and intestine. Measurements of nasal potential difference in H+/m− mice were not significantly different from WT mice suggesting substantial hCFTR function in the airway. Further studies of the intestine showed evidence of bicarbonate transport and sensitivity to CFTR_inh_-172. Measurements of small intestine I_sc_ in H+/m− mice demonstrated a smaller response to cAMP stimulation in comparison to mice expressing endogenous mCftr. With respect to the small intestinal responses, the H+/m− ΔI_sc_ upon cAMP stimulation (~60–80 µA/cm²) is within the range of cAMP-stimulated ΔI_sc_ previously reported for control human small intestine (~25–90 µA/cm²)^35–38^, although greater responses have since been observed^39^. Importantly, the cAMP-stimulated ΔI_sc_ by the H+/m− colon (~160–170 µA/cm²), as well as CFTR_inh_-172 inhibition, were similar in magnitude to the endogenous mCftr colonic responses and matched well with previously reported cAMP-stimulated ΔI_sc_ of control human colon (~85–145 µA/cm²)^36,40^. Overall, electrophysiological studies of H+/m− intestine show that the I_sc_ responses are perhaps more consistent with human than murine intestine and that the colonic mucosa may provide the best signal-to-noise ratio for discerning I_sc_ differences between CFTR modulators.

One limitation with the hCFTR model is the integration of multiple copies of the BAC at the single integration site. The integration of multiple copies arranged as a concatemer is common in microinjected DNA including BACs^41,42^. Our data suggest that there are six copies of hCFTR at this single site on mouse chromosome 8. Using traditional gene editing methods, inserting human CFTR mutations into these multiple copies of hCFTR may prove very difficult. However, utilizing CRISPR/Cas9 this feat may now be achieved with several reports citing gene editing of multiple genes at one time at high frequencies^43–45^. Future experiments to insert common human CFTR mutations in this model using CRISPR/Cas9 are necessary to determine the level of difficulty for editing the six hCFTR copies in this model.

A BAC transgenic mouse expressing hCFTR and its major regulatory elements has been developed and shows robust CFTR function in the airway and intestinal mucosa. The morphological and functional rescue by the BAC transgene in Cftr KO mice provides evidence of sufficient hCFTR activity at the apical membrane of CF-relevant epithelia. Thus, the opportunity presents itself to create mouse models expressing CF-causing mutations by gene editing methods. The functional activity of hCFTR in the BAC transgenic mouse suggests that introduction of disease-causing mutations will provide animal models useful for *in vivo* screening of CFTR potentiators such as VX-770. On the other hand, the response to CFTR processing correctors such as VX-890 may be more difficult to interpret because of the increased background of poorly processed hCFTR. An alternate scenario is that the sensitivity to identify processing correctors will be improved by greater availability of immature mutant hCFTR. Investigation of mutant hCFTR expression in the BAC transgenic mouse will likely increase our understanding of the biology behind CFTR processing. Overall, the generation of BAC transgenic mice carrying medically relevant hCFTR mutations is the next step in providing pre-clinical models for *in vivo* testing of CFTR modulators and other therapies for CF disease.

## Methods

### Mice

To generate hCFTR transgenic mice, a 250.3 kb BAC DNA fragment containing the entire coding sequence of hCFTR with its 27 exons, 40.1 kb of DNA 5′ and 25 kb of DNA 3′ to the gene was linearized using a Not1 digest of CFTR1,2,3OE BAC and microinjected into the male pronuclei of C57/BJ zygotes (Victor Lin, Precision Targeting, Inc., Tenafly, NJ). hCFTR founder mice were identified by PCR of genomic DNA using the following primers^46^: BAC123s-gDNA-5′ set (Exon/Intron 1): 5′-AGCGCCCGAGAGACCATGCA-3′/5′-CGAAGCTCGGTTGGCCACCTT-3′; BAC123s-gDNA-Mid set (Intron 10): 5′-GGGGGCCATGGGACTCACTGA-3′/5′-TGCCACCAGCCTTACCGCTC-3′; and BAC123s-gDNA-3′ set (Intron 20): 5′-CACCGGCATTCCCCAATGAGACAG-3′/5′-TGCATGGAGCATTGCACAGCTGTTA-3′. hCFTR founders were outbred to Black Swiss (Charles River) mice and offspring were tested for expression of the hCFTR to assess germ-line transmission. The integrity of the transgene mRNA from the offspring of founders was probed using a TaqMan® assay (Life Sciences) designed to cross the exon 27–28 boundary near the C-terminus of hCFTR. Founder offspring expressing the BAC transgene were bred to mice with gene targeted disruptions of the murine homolog of *Cftr* [*abcc7*, Cftr knock-out (KO)]^27^ to generate mice that were positive for *hCFTR* and *mcftr*−/− (H+/m−) or negative for *hCFTR* and *mcftr*−/− (H−/m−). Survival, growth, histopathology or electrophysiological parameters were not different in mice expressing *mcftr* (*mcftr*+/+ or *mcftr*+/−) and that were either positive or negative for *hCFTR*, so these groups were combined as wild-type (WT). Genotypes were identified by PCR analysis of tail-snip DNA as previously described^47^. All mice were maintained *ad libitum* on standard laboratory chow (Formulab 5008, Rodent Chow; Ralston Purina) and distilled water in survival/growth experiments or distilled water containing Colyte® (Schwartz Pharma) laxative to prevent intestinal obstruction in the H−/m− mice^47^. Mice were housed individually in a temperature- and light-controlled room (22–26 °C; 12-hour light: 12-hour dark cycle) in the Association for Assessment and Accreditation of Laboratory Animal Care-accredited animal facility at the Dalton Cardiovascular Research Center, University of Missouri. Mouse experiments were performed in accordance with guidelines outlined in “Guide for the Care and Use of Laboratory Animals” prepared by the National Academy of Sciences and published by the National Institutes of Health and with approval from the University of Missouri Animal Care and Use Committee as well as the Case Western Reserve University Animal Care and Use Committee.

### Histology

Jejunal sections were fixed in 10% neutral buffered formalin, dehydrated, and embedded in paraffin. Samples were stained with hematoxylin and eosin and observed using light microscopy. For measurements of crypt and villus length, samples from five mice per genotype were scanned via a Leica SCN400 slide scanner and images were analyzed via VisiomorphDP software (Visiopharm).

### Immunoblot analysis

Freshly isolated murine jejunal crypts, CHO cells transfected with human CFTR (gift of T.-C. Hwang, University of Missouri) and non-transfected CHO cells were suspended in ice-cold PBS or RIPA containing Halt™ Protease inhibitor (ThermoFisher) and lysed at 4 °C by supersonication. Samples were centrifuged at 13, 000 rpm for 12 min at 4 °C. In some cases, supernatant was treated with PNGase F (New England BioLabs) for four hrs. at 37 °C then loaded on SDS-PAGE gels for electrophoresis, membrane transfer and immunoblotting. Anti-Cftr 3g11 (1:500 dilution, provided by CFTR Folding Consortium) was used as primary antibody. Secondary antibody was goat pAb to rat IgG (1: 2000 dilution, abcam, ab7097). Anti-GAPDH (1:2000 dilution, abcam, ab9485) was used as loading control and secondary antibody was goat pAb to rabbit IgG (1:2000 dilution, abcam, ab6721).

### Reverse transcription and quantitative PCR array (qRT-PCR)

Tissue samples (duodenum, jejunum, ileum, cecum, proximal colon, distal colon, nasal mucosa, trachea, lung, spleen, heart, testes, uterus, liver, pancreas, salivary gland and kidney) were stored in RNA Later (−80 °C) until processing. Tissues were homogenized and total RNA extraction was performed using RNeasy Plus (according to the manufacturer’s protocol, Qiagen). Isolated RNA was reverse transcribed with Superscript III First Strand Synthesis Kit (Invitrogen) using oligo dT according to the manufacturer’s protocol. cDNA was used with individual TaqMan® Gene Expression Assays (ThermoFisher), according to the manufacturer’s protocol, for the exon 27–28 boundary near the C-terminus of hCFTR (Assay ID Hs01565549_m1) and three housekeeping genes β-glucuronidase (Assay ID Mm00446953_m1), hypoxanthine guanine phosphoribosyl transferase 1 (Assay ID Mm00446968_m1), mitochondrial ribosomal protein L19 (Assay ID Mm00452754_m1).

### Enteroid culture and videomicroscopy

The enteroid culture of isolated crypt epithelium from the jejunum has been previously described in detail^48^. Distal jejunal enteroids were plated in chambered glass microscope slides (ThermoFisher) and overlaid with growth medium containing Ham’s F-12 medium with 5% FBS, 50 µg/mL gentamicin, 125ng/mL R-spondin1, 25 ng/mL noggin and 12.5 ng/mL epidermal growth factor. Growth medium was changed every 3–4 days and enteroids were passaged every 7–10 days. Passages 1–2 were used for experimentation. For videomicroscopy, the microscope slide was fitted with a polycarbonate perfusion chamber (Warner Instruments) for superfusion at 37 °C. Single goblet cells were imaged in cross-section using a 60x water immersion objective of an upright Olympus BX-50WI microscope (Olympus). Enteroids were luminally perfused with a micropipette for ~5-min before the addition of 100 µM carbachol into the basolateral bath to stimulate exocytosis. Images were acquired at 5-sec intervals for ~15 min using a Sensi-Cam digital camera (Cooke) and Slidebook 5.0 software (Intelligent Imaging Innovations).

### Nasal potential difference (NPD) measurements

NPDs were obtained from the nasal lumen in anesthetized mice as previously described^49,50^. The change in NPD (mV) was assessed in response to chloride-free HEPES-buffered Ringer’s containing forskolin (10 µM) and amiloride (100 µM).

### Identification of hCFTR BAC integration site and copy number

The hCFTR BAC insertion site in the mouse was identified using Mate Pair whole genome sequencing on an Illumina HiSeq instrument. The resulting data was mapped using CLC Genomics Workbench 8.02 from Qiagen and used to identify junction sequences between the transgene sequences and the mouse genome (GRCm38-mm10). In addition, fluorescence *in situ* hybridization (FISH) was completed using a fluorescently labeled BAC on mouse embryonic stem cells created from embryos carrying the hCFTR transgene by Cell Line Genetics as previously described^51^. A single insertion site on mouse chromosome 8 was identified in region 8qB2 (58490672 to 58501953). At this single integration site, the copy number of hCFTR was estimated using qPCR with 3 sets of primers spread throughout hCFTR as previously described^52^.

### Short-circuit current measurement

Freshly excised intestinal sections were stripped of the seromuscular layers and mounted on Ussing chambers for the measurement of short-circuit current (I_sc_) as previously described^21^. The mucosal surfaces were bathed with Krebs bicarbonate Ringers (KBR) containing (in mM): 140.0 Na^+^, 5.2 K^+^, 2.8 PO_4_^2−^, 115.0 Cl^−^, 25.0 HCO_3_^−^, 1.2 Ca^2+^, 1.2 Mg^2+^, 4.8 gluconate^−^, 10.0 mannitol (apical) or glucose (basolateral) and gassed with 95% O_2_-5% CO_2_ at 37 °C (pH 7.4). KBR baths contained 1 µM indomethacin (bilateral) and 0.1 µM tetrodotoxin (basolateral). In Cl^–^free experiments, 115 mM Na^+^ isethionate^−^ was substituted equimolar for NaCl. Colonic preparations were pre-treated with 10 µM amiloride to block electrogenic Na^+^ absorption. Following a ~20-min equilibration period, intestinal tissue were treated with a forskolin (10 µM)/3-isobutyl-1-methylxanthine (100 µM, IBMX) cocktail for 20 min before treatment with CFTR_inh_-172 (10 µM, 15 min).

### Materials

EGF and noggin were obtained from R&D Systems (Minneapolis, MN). Recombinant R-spondin1 was isolated as described previously^53^.

### Equipment and settings

Tissue photomicrographs (H&E stain) - Images acquired using Olympus IMT2 inverted light microscope (20X objective), an Infinity 1–3 C camera (Lumenera) and Image Pro Plus 6.3 software (Media Cybernetics) at pixel dimension = 2048 × 1536, image bit depth = 24 and resolution 300dpi (HxV). Images were adjusted equally for brightness and contrast. Goblet cell videomicroscopy – Images were acquired using an Olympus BX-50WI upright light microscope (60X objective), a Sensi-Cam camera (Cooke) and Slidebook 5.0 software (Intelligent Imaging Innovations) at pixel dimension = 640 × 512, image bit depth = 24 and resolution = 96dpi (HxV). Images were adjusted for brightness and contrast. Immunoblots – Images were acquired using ChemiDoc XRS + System and ImageLab 5.1 software (BioRad) at pixel dimension = 3921 × 2924, image bit depth = 24 and resolution = 600dpi (HxV).

### Imaging flow cytometry

Lung tissue was isolated from mice and passed through a 70 µm cell strainer. Cells were resuspended in 1x PBS with a Complete Protease inhibitor tablet (Roche). Separated cells were kept on ice and fixed for 30 minutes with 4% paraformaldehyde at 4 °C. All staining procedures were carried out in the presence of 2% BSA in 1x PBS. Assessment and quantification of CFTR were performed using imaging flow cytometry with RB7865, a rabbit polyclonal antibody directed towards the first extracellular loop of CFTR (Biomatik) (1). Primary anti-CFTR antibody RB7685 was adjusted to the same final protein concentration of 240 ng/µl, AF488 secondary antibody was prepared at 1:5000 dilution according to the manufacturer’s instructions^54^. For each sample, 20000 events were collected on the Amnis Imagestream using Channels 1(Brightfield1), 2 (AF488), 6 (Sidescatter), 9(Brightfield2) and 11(DRAQ5). The following laser powers were used throughout the experiment, 488 nm = 100 mW, 658 nm = 120 mW and 768 nm = 3.52 mW. The single dye compensation controls were collected at the same laser powers to adjust for bleed over between channels. Data processing was carried out using IDEAS 6.2. All samples were processed with identical settings, first focused cells were selected and then divided into single cells and multiple cells using the Area versus Aspect Ratio discrimination. The single cell population was then further evaluated by the length in the brightfield to identify ciliated cells. To further discriminate between dead and live cells the distribution of the nuclear stain versus the CFTR stain was evaluated. Cells with co-localization of CFTR and DRAQ5 were considered dead or permeabilized. The remaining cell population was then used for quantification and image analysis.

### Statistics

Cumulative data are reported as the mean ± SE. Data between two groups were compared using a two-tailed unpaired Student *t*-test or, if not normally distributed with equal variances, by Mann-Whitney Rank Sum test. Data from multiple treatment groups were compared using a one-way ANOVA with a post hoc Tukey’s *t*-test. Data from the I_sc_ time course were tested using repeated measures ANOVA with a Dunnet’s t test. The non-parametric Kaplan-Meier estimator test was used to determine differences in survival. The Kruskal-Wallis test was used to determine differences between cell populations with CFTR antibody staining. A probability value of *P* < 0.05 was considered statistically significant.

## Data Availability

The authors will make materials, data and associated protocols promptly available to readers without undue qualifications in material transfer agreements.
